# Z-BeEAM (Ibritumomab tiuxetan, Bendamustine, Etoposide, Cytarabine, Melphalan) before autologous stem cell transplantation is safe and efficient for refractory large B-cell lymphoma

**DOI:** 10.1186/s40164-015-0013-2

**Published:** 2015-07-04

**Authors:** Magalie P. Tardy, Lauris Gastaud, Mario Ojeda-Uribe, Annick Boscagli, Salvatore Caruso, Richard Skaf, Jean Gutnecht, Antoine Thyss, Frédéric Peyrade

**Affiliations:** Department of Oncology, Antoine-Lacassagne Center, Nice, France; Department of Hematology, Mulhouse Hospital, Mulhouse, France; La Dracenie Hospital Center, Draguignan, France; Department of Oncology, Saint-Georges Clinic, Nice, France; Department of Oncology, Frejus-Saint Raphael Hospital, Frejus, France

**Keywords:** Aggressive Non-Hodgkin lymphoma, Large B cell lymphoma, Autologous stem cell transplantation, Conditionning regimen, Z-BeEAM

## Abstract

**Background:**

Refractory or relapsed large B-cells lymphoma are usually treated with a high dose chemotherapy regimen followed by an autolougous stem cells transplantation. BEAM (carmustine, etoposide, cytarabine, melphalan) or more recently Z-BEAM (ibritumomab tiuxetan and BEAM) are commonly used regimens, but recently carmustine availability became difficult. The purpose of this study was to evaluate the feasibility and the safety of replacing carmustine by bendamustine in a new Z-BeEAM regimen (ibritumomab tiuxetan, bendamustine, etoposide, cytarabine, melphalan) prior to autologous stem cell transplantation.

**Findings:**

This study was a retrospective analyze of six patients, with a median age of 60, treated by Z-BeEAM before autologous stem cell transplantation. We did not put in evidence any additional toxicities compared to conventional induction chemotherapy. The main toxicities were mucositis (3 grade III among 6 patients), gastrointestinal (2 grade III vomiting and 2 grade III diarrhea) and neutropenia (6 grade IV). Engraftment was successfully achieved for all patients. At the time of analysis of this study all patients were alive and in complete response based on the PET-CT evaluation.

**Conclusions:**

BeEAM plus ibritumomab tiuxetan combined regimen before autologous stem cell transplantation is feasible and safe in aggressive relapsing large B-cell lymphoma.

## Introduction

Diffuse large B-cell lymphoma (DLBCL) is usually associated with good prognosis. Two-thirds of patients will be cured after classical chemotherapy (R-CHOP or R-ACVBP). However, for relapsing or refractory aggressive DLBCL, high-dose chemotherapy (HDT) followed by autologous stem cell transplantation (ASCT) is the standard treatment if the patient is able to tolerate this intensive protocol [1, 2].

BEAM (carmustine, etoposide, cytarabine, melphalan) is the most commonly used chemotherapy for intensification prior to ASCT [3]. Recently, it has been proven that adding yttrium-90 ibritumomab tiuxetan (Zevalin; Spectrum Pharmaceuticals, Irvine, CA), a radiolabeled anti-CD20 monoclonal antibody, to BEAM is safe and increases both progression-free survival (PFS) and overall survival (OS) in patients with DLBCL [4]. The 2-year OS was 91 % in the Z-BEAM group compared to 62 % in the BEAM group (p = 0.05), with no significant additional toxicity.

In recent months, there has been a supply problem regarding carmustine, used in the BEAM protocol [5]. Some teams involved in ASCT have tried to replace carmustine with bendamustine, another alkylating agent, which also has the antimetabolite activity of the purine analog structure. A phase I-II study showed the safety and efficacy of bendamustine coupled with etoposide, cytarabine and melphalan (BeEAM) in the conditioning regimen to ASCT for refractory or relapsed DLBCL. 72 % of patients with resistant lymphoma who underwent BeEAM regimen prior to ASCT were still in complete remission 3 years after treatment [6].

This present study demonstrates the feasibility and safety of high dose chemotherapy using the Z-BeEAM regimen with a retrospective analysis of 6 patients treated for relapsed lymphoma.

## Material and methods

### Patient population

All patients with CD20^+^-aggressive non-Hodgkin lymphoma treated with a Z-BeEAM conditioning regimen prior to ASCT at the Centre Antoine Lacassagne, Nice, and at the Centre Hospitalier, Mulhouse, France, were retrospectively analyzed. Six patients received this salvage therapy between August 2014 and March 2015. Characteristics of patients are presented in Table 1.

**Table 1:** Patients and disease characteristics

Characteristics	Number of patients
Median age : years (range)	60 (51–66)
Gender	
- Male	5
- Female	1
Performans Status (PS)	
- 0	6
- >0	0
Disease stage	
- ≤2	0
- >3	6
Number of previous chemotherapies	
- 1	1
- 2	3
- 3	2
Disease status at transplantation	
- Complete response	3
- Partial response	3
PET-CT before transplantation	
- Negative	3
- Positive	3
Median time between diagnosis or relapse and ASCT (months) (range)	8.5 (7–12)

Before treatment, all patients were evaluated with a clinical examination, echocardiography, abdominal echography and biologic and serologic tests. Response to chemotherapy was evaluated before ASCT by positron-emission tomography with computed tomography (PET-CT).

### Collection and processing of progenitor cells

Peripheral blood stem cells were collected either during recovery after an induction chemotherapy cycle, or during a specific mobilization procedure using cyclophosphamide (1.5 g/m^2^). Between one and three leukaphereses were performed after stimulation using filgrastim 5 μg/kg/12H to obtain at least 2 million CD34^+^ cells/kg. When insufficient circulating CD34^+^ cells were obtained with filgrastim, leukapheresis was performed after plerixafor injection.

### Conditioning regimen

The day of peripheral-blood stem cell reinjection, performed as a direct venous infusion, was considered as day 0. The conditioning regimen started effectively on day -21 with an injection of rituximab (250 mg/m^2^), followed on day -14 by a second injection of rituximab and an injection of ibritumomab (1200UI). Patients were then hospitalized and, from day -7 to day -1, received the BeEAM regimen consisting of bendamustine (day -7 at 180 mg/m^2^), etoposide and cytarabine (from day -6 to day -2 at 200 mg/m^2^ every 12 h), melphalan (140 mg/m^2^) and amifostine (740 mg/m^2^) on day -1.

### Supportive care and clinical monitoring

All patients were hospitalized in single rooms in reverse isolation and received antimicrobial prophylaxis until neutrophil recovery with ciprofloxacine (500 mg per os every 12H), fluconazole (200 mg intravenously every 12H) and oral decontamination using diluted vancomycin. Broad-spectrum antibiotics (ceftazidime) were given in case of febrile neutropenia. When invasive fungal infection or fever lasted more than 5 days, an antifungal (voriconazole) was added. Hematopoietic growth factors (filgrastim 5 μg/kg/day) were given from day 5 to the end of aplasia. Platelet transfusion was performed when the platelet count was <10 × 10^9^/L, <20 × 10^9^/L in case of fever or bleeding and <50 × 10^9^/L in case of disseminated intravascular coagulation. Packed RBCs were administered to maintain a hemoglobin level >8 g/dl. Patients underwent daily physical examination and blood test during hospitalization. Cotrimoxazole and acyclovir were then administered for 6 months after ASCT to prevent pneumocystis jirovecii and viral infections.

### Data collection and objectives

All adverse events occurring during the treatment period were graded using the Common Terminology Criteria for Adverse Events (CTCAE version 4.0). Each patient underwent a CT-Scan and a PET-CT three months after ASCT to assess response (complete remission or partial response) [7]. The main objective of this study was to analyze the safety of the Z-BeEAM protocol used as high-dose chemotherapy followed by ASCT, considering the incidence and the grade of early adverse effects.

## Results

### Patients and treatment characteristics

There were 5 males and 1 female, with a median age of 60 years (range 51–66). All received at least one rituximab-containing chemotherapy before salvage therapy. One of the six patients had already received a first ASCT in 2008 and relapsed in 2014. Five patients had at least 2 different lines of chemotherapy before HDT and ASCT, and one also had radiotherapy in addition to chemotherapy. Two patients had a mantle lymphoma, three had a diffuse large cell lymphoma, and one had a follicular lymphoma. All were PS 0 at the time of ASCT.

Three patients were in complete response when they underwent ASCT, and 3 in partial response. Median time between diagnosis or relapse and ASCT was 8.5 months (7–13).

The mean number of reinjected CD34^+^ cells was 2,165.10^6^/kg (Table 2). Stem cell mobilization was achieved with G-CSF alone in 3 cases. Two other patients needed adjunction of perixafor and one required the use of cyclophosphamide (1.5 g/m^2^).

**Table 2:** Engraftment characteristics

Engraftment characteristics	Values (range)
Median number of CD34^+^ reinfused	2,165. 10^6^
Median time to PNN > 0.5. 10^6^ (days)	12 (9–23)
Median time to reach aplasia (days)	7 (0–9)
Median days to hospital discharge (days)	26 (20–26)
Number of CGR transfusions	2 (0–6)
Number of CPA transfusions	6 (3–13)

### Toxicity

Early toxicity data are presented in Table 3. All patients presented grade IV neutropenia, reached in a median time of 7 days after the beginning of chemotherapy, except for one patient who presented neutropenia before starting chemotherapy. The median time to obtain an absolute neutrophil count >0.5 × 10^9^/L was 12 days. Engraftment was successfully achieved in all patients. All patients experienced at least grade III thrombopenia and anemia with a median need of 6 platelet transfusions (3–13) and 2 red blood cell transfusions (0–6) (Table 2). One of the other major side-effects observed was mucositis; 3 patients presented grade III mucositis requiring morphinic treatment, one patient presented grade II mucositis and 2 had grade I. Gastrointestinal toxicities were frequently found with 2 patients presenting grade III diarrhea and 2 grade III vomiting. All patients presented fever during their neutropenia period requiring empiric antibiotherapy and antifungal therapy was added in one case. These febrile neutropenias were all easily managed with these treatments and none have evolved to septic shock. No patients died during the treatment.

**Table 3:** Non-hematologic early toxicities

Toxicity	All grades (number of patients)	Grade I-II	Grade III	Grade IV
Mucositis	6	3	3	0
Gastro-intestinal				
- Nausea/vomititis	3	1	2	0
- Diarrhea	5	3	2	0
Fever	6	5	1	0
Documented infection	1			
Cardiac arrythmia	0			
Pulmonary toxicities (pneumonitis)	0			

### Response to treatment

The mean follow-up period was 5 months (0–10). At the end-point (1^st^ March 2015), all patients were alive and in complete response based on clinical examination, biological examination and on the PET-CT results.

## Discussion

High-dose chemotherapy followed by ASCT is the standard treatment for refractory or relapsed aggressive DLBCL [2], usually reserved for young (<65 years) and fit patients. Various conditioning regimens have been used prior to ASCT with no evidence that one is superior to the others. However, the BEAM regimen is the most widely used due to its limited morbidity and toxicities, and with comparable efficacy [8, 9]. It has also been proven that the BEAM regimen is effective and safe for selectionned elderly patients [10] with a good performans status.

However, the CORAL study [1] demonstrated that patients with relapsed DLBCL, previously treated with rituximab, do very poorly after ASCT. Since rituximab is now widely used, it has become necessary to develop novel approaches to enhance these results. One of them has been the addition of radioimmunotherapy as part of the conditioning regimen prior to autologous stem cell transplantation [11]. Fruchart *et al.* [4] studied the association of ibritumomab and the BEAM regimen prior to ASCT for patients receiving a first rituximab-containing chemotherapy. They observed an overall response rate of 86 %, and a 2-year event-free survival of 79 %. Furthermore, it is safe to add ibritumomab to the BEAM regimen with no increase in toxicity except for mucositis with a majority of grade III [12]. The results of our small retrospective study show that no additional toxicities are observed when adding ibritumomab to the BeEAM protocol. The duration of grade IV neutropenia is similar to that described in the majority of studies using a classical BEAM regimen [13]. There was no difference in engraftment compared with other studies and transfusion needs were similar to those previously reported with BEAM, BeEAM or Z-BEAM regimens. The principal toxicities noted were gastrointestinal toxicity and mucositis. This was expected as similar studies using radioimmunotherapy revealed same type and level of toxicity.

The main limitation of our study is the small number of patients and the short follow-up period which has not allowed us to explore long-term response and delayed toxicity. It would be interesting to search for late cardiac and hematologic toxicity, especially myelodysplasic syndrome. Another important question would be the strategy to adopt in patients relapsing after treatment containing rituximab, ibritumomab and BeEAM prior to ASCT.

BeEAM (bendamustine, etoposide, cytarabine, melphalan) plus yttrium-90 ibritumomab tiuxetan combined regimen before autologous stem cell transplantation is feasible in aggressive relapsing large B-cell lymphoma. Further prospective clinical trials are needed to confirm our preliminary results.

## Abbreviations

ASCT: autologous stem cell transplantation
BEAM: Carmustine, Etoposide, Cytarabine, Melphalan
BeEAM: Bendamustine, Etoposide, Cytarabine, Melphalan
DLBCL: diffuse large B cell lymphoma
HDT: high dose (chemo)therapy
OS: overall survival
PFS: progression-free survival
